# Modulatory Effects of Alpha-Mangostin Mediated by SIRT1/3-FOXO3a Pathway in Oxidative Stress-Induced Neuronal Cells

**DOI:** 10.3389/fnut.2021.714463

**Published:** 2022-01-28

**Authors:** Waralee Ruankham, Wilasinee Suwanjang, Kamonrat Phopin, Napat Songtawee, Virapong Prachayasittikul, Supaluk Prachayasittikul

**Affiliations:** ^1^Faculty of Medical Technology, Center for Research and Innovation, Mahidol University, Bangkok, Thailand; ^2^Department of Clinical Microbiology and Applied Technology, Faculty of Medical Technology, Mahidol University, Bangkok, Thailand; ^3^Department of Clinical Chemistry, Faculty of Medical Technology, Mahidol University, Bangkok, Thailand; ^4^Faculty of Medical Technology, Center of Data Mining and Biomedical Informatics, Mahidol University, Bangkok, Thailand

**Keywords:** alpha-mangostin, antioxidant, oxidative stress, sirtuin, Alzheimer's disease

## Abstract

**Background:**

alpha-Mangostin, a polyphenolic xanthone, is primarily found in the pericarp of mangosteen throughout Southeast Asia and is considered as the “Queen of Fruit” in Thailand. Nonetheless, it is not clarified how alpha-mangostin protects neuronal cells against oxidative stress.

**Objective:**

In this study, molecular mechanisms underlying the neuroprotective effect of alpha-mangostin in defending hydrogen peroxide (H_2_O_2_)-induced neurotoxicity was explored.

**Methods:**

cytotoxicity, reactive oxygen species (ROS) generation, apoptotic cascades, and protein expression profiles were performed incorporation of molecular docking.

**Results:**

Human SH-SY5Y cells were pretreated with 1 μM alpha-mangostin for 3 h prior to exposure to 400 μM H_2_O_2_. alpha-Mangostin significantly inhibited oxidative stress-induced cell death in neuronal cells by reducing BAX protein, decreasing caspase-3/7 activation, and increasing anti-apoptotic BCL-2 protein. Collectively, alpha-mangostin was demonstrated to be a prominent ROS suppressor which reversed the reduction of antioxidant enzymes (CAT and SOD2). Surprisingly, alpha-mangostin significantly promoted the expression of the sirtuin family and the FOXO3a transcription factor exerting beneficial effects on cell survival and longevity. A molecular docking study predicted that alpha-mangostin is directly bound to the active site of SIRT1.

**Conclusion:**

Findings from this study suggest that alpha-mangostin potentially serves as a promising therapeutic compound against oxidative stress by activation of the SIRT1/3-FOXO3a pathway comparable to the effect of memantine, an anti-AD drug used for the treatment of moderate to severe dementia.

## Introduction

Oxidative stress is characterized by the over-accumulation of free radicals that disrupt equilibrium in antioxidant homeostasis. The formation of free radicals generated by redox metals has potential roles in regulating many cellular processes, especially reactive oxygen species (ROS) in progressive deterioration of neurons (1, 2). Age-related neurodegenerative disorders including Alzheimer's disease (AD), Parkinson's disease (PD), Huntington's disease (HD), and Amyotrophic lateral sclerosis (ALS) affect millions of people worldwide. Particularly, AD has been documented to be in the top 10 leading cause of death among people age 65 and older. As the proportion of older people is increasing, the number of aging population living with Alzheimer's or other types of dementia will grow rapidly up to 139 million by 2050 (3). These can cause serious problems with emotional and behavioral symptoms such as memory loss, mental illness, and physical inactivity.

More effective neurodegenerative therapeutics are urgently needed for development. Antioxidant therapies are promising alternatives for prevention or treatment due to their ability to scavenge free radicals. These hypotheses provide a rationale for the assessment of antioxidant therapy in *in vitro* and *in vivo* models of neurodegeneration. Mangosteen (*Garcinia mangostana* Linn.) is categorized in the *Guttiferae* family found in tropical regions, particularly in Thailand, Myanmar, India, Malaysia, and Indonesia. The pericarp of mangosteen is a rich source of phenolic compounds which function as natural antioxidants, including phenolic acids, xanthones, anthocyanins, and oligomeric procyanidins (4, 5). These are effective nutrients in the prevention of oxidative degradation and/or act as radical scavengers (6). alpha-Mangostin, a polyphenolic xanthone substituted by hydroxyl, methoxy, oxo, and prenyl groups, is primarily present in the pericarp of mangosteen. It displays diverse biological properties such as antibacterial (7–11), anticancer (12), anti-inflammatory (13), as well as prevention of hepatic steatosis (14) and obesity (15). Increasing attention of antioxidant strategy is being paid, not to the treatment of the neurodegenerative patients, but in a more prevention and improvement to the life extension.

One of the longevity factor which have been reported to be involved in neuroprotection is sirtuin family. Sirtuin (SIRT) is a class III protein deacetylase that has been well studied in many biological processes. There are 7 members (SIRT1-7) of this family which target and localize in the different parts of the cell. SIRT1 and SIRT3 exert these potential influence through acetylation of essential neuroprotective proteins, i.e., peroxisome proliferator-activated receptor γ co-activator-1α (PGC-1α), p53, Mammalian target of rapamycin (mTOR), and nuclear factor kappa B (NF-κB) (16). Additionally, the activation of forkhead box O3a (FoxO3a) in neurons regulates downstream targets of SIRT1 and SIRT3 in several molecular pathways including neuronal survival, mitochondrial homeostasis, and antioxidant balance (17). The major sources of cellular ROS are mitochondria. Therefore, an impairment of mitochondria significantly contributes to the production of ROS and oxidative stress that are involved in aging. The production of ROS influenced by neurodegenerative processes might affect mitochondrial parameters as well as activation of mitochondrial permeability transition pore (MPTP), membrane potential (ΔΨm), calcium (Ca^2+^) influx, and ATP production that result in neuronal damage (18, 19). ROS are extremely active in mitochondrial damage and stimulate the apoptotic cascade by damaging other cellular components. These include proteins that lead to loss of enzyme activity which regulates oxidative balance in the cellular system such as catalase (CAT), superoxide dismutase (SOD), and glutathione peroxidase (GPx) (20, 21). A recent review proposed that these SIRT1 and SIRT3 proteins might be the next targets for intensive study because of their potential associated with neurodegenerative diseases.

Therefore, alpha-mangostin may modulate neurodegeneration by any of multiple mechanisms, and this is the subject of an ongoing investigation. To achieve a better understanding of the neuroprotective capacity of alpha-mangostin, the effect of the compound on H_2_O_2_-induced neurotoxicity in human neuronal cells was explored and compared with memantine, an N-methyl-D-aspartic receptor (NMDAR) antagonist for clinical use in AD patients.

## Materials and Methods

### Chemicals and Reagents

alpha-Mangostin (≥98% purity) and memantine hydrochloride (≥98%) were purchased from Sigma Aldrich (St Louis, MO, USA). Hydrogen peroxide (H_2_O_2_, 30%), Annexin V & Dead Cell Assay Kit, and Muse™ Caspase-3/7 Kit were obtained from Merck Millipore (Darmstadt, Germany). 3-(4,5-Dimethylthiazol-2-yl)-2,5-diphenyltetrazolium bromide (MTT) and 2′,7′-dichlorodihydrofluorescin diacetate (H_2_DCFDA) were purchased from Molecular Probes (Eugene, Oregon, USA). Monoclonal antibodies against BAX, BCL-2, SIRT1, SIRT3, FOXO3a, CAT, SOD2, and actin, as well as horseradish peroxidase (HRP)-conjugated goat anti-mouse and rabbit secondary antibodies, were purchased from Cell Signaling Technology (Beverly, MA, USA). Enhanced chemiluminescence (ECL) Plus Western blotting reagent was supplied from Amersham Biosciences (Piscataway, NJ, USA). All other chemicals and reagents were of analytical grade from Sigma Aldrich (St Louis, MO, USA).

### Cell Culture

Human SH-SY5Y neuroblastoma cells originated from the American Type Culture Collection (ATCC) (Manassas, VA, USA) were routinely maintained in Dulbecco's Modified Eagle's Medium (DMEM) supplemented with 10% fetal bovine serum (FBS) and 1% penicillin-streptomycin from Gibco BRL (Gaithersburg, MD, USA) in a humidified atmosphere (37°C) containing 95% air and 5% CO_2_.

### MTT Assay

**Figure 1:**
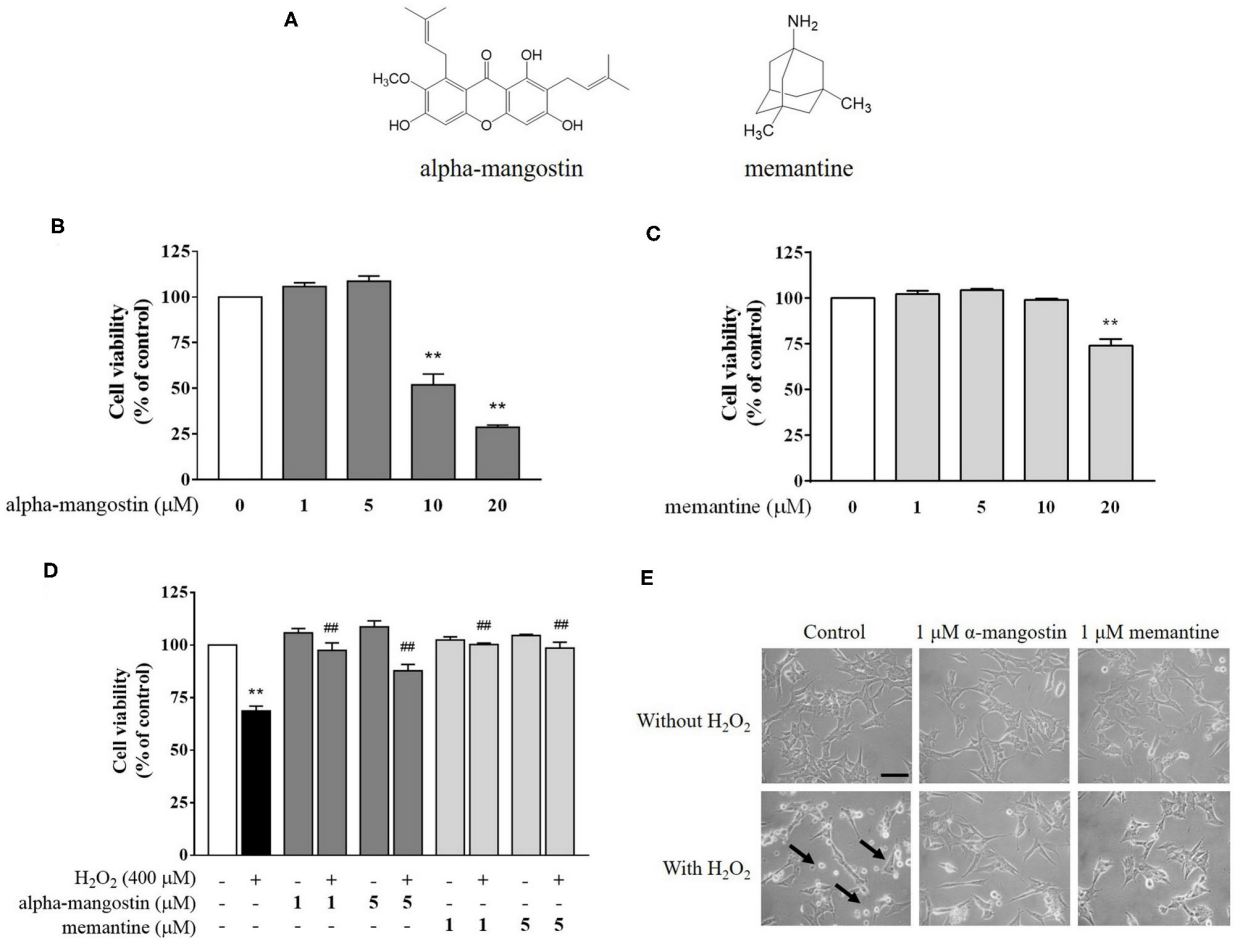
**(A)** Chemical structures of alpha-mangostin and memantine. **(B–D)** Protective effects of alpha-mangostin and memantine on H_2_O_2_-induced cell viability in SH-SY5Y cells. The cell viability was assessed with MTT assay. **(E)** Phase-contrast images of SH-SY5Y cells pretreated with alpha-mangostin and memantine at 40x magnification. The arrows indicate typically round-shaped cells, shrinkage, and no longer adherent. Data are presented as mean ± SEM of four independent experiments. **P < 0.01 vs. control and ^*##*^*P* < 0.01 vs. H_2_O_2_.

A reduction of MTT to colored formazan is widely used to assess cellular metabolic activity. To investigate the influence of alpha-mangostin on cytotoxicity in neuronal SH-SY5Y cells, the cells were seeded into 96-well culture plates at a density of 1x10^5^ cells/mL and placed in an incubator for 24 h. Following the incubation, the cells were treated with various concentrations of alpha-mangostin (1, 5, 10, and 20 μM) or memantine (the drug used for AD) for 3 h prior to treatment with 400 μM H_2_O_2_ for a further 24 h (Figure 1A). The MTT in 0.1 mM phosphate buffer saline (PBS) was added to each well and incubated at 37°C for 3 h. The solution was replaced by the extraction buffer (0.04N HCl-isopropanol). The 96-well plates were analyzed on a microplate reader (BioTek Instruments, VT, USA) at 570 nm spectral wavelength.

### Annexin V Apoptosis and Caspase-3/7 Activation Assays

Annexin V/7-AAD was adopted to identify all stages of apoptosis by flow cytometry. Caspase is one of the protease enzymes that plays an important role in apoptotic processes, especially caspase-3 and caspase-7 as downstream targets in the apoptotic signaling cascades. Briefly, the SH-SY5Y cells were seeded into 6-well culture plates at a density of 1x10^5^ cells/mL and then incubated at 37°C overnight. The culture medium was replaced by complete medium containing 1 μM alpha-mangostin or memantine for 3 h, followed by a further incubation with 400 μM H_2_O_2_. After 24 h of treatment, the cells were washed with PBS once to remove unbound particles, trypsinized, and then centrifuged at 25 °C with 1,500 rpm for 5 min. The pellet was resuspended in complete growth medium and finally subjected to Muse™ cell analyzer.

### DCFDA Assay

Intracellular ROS level was measured by 2′,7′-dichlorofluorescin diacetate (H_2_DCFDA) which reacted with intracellular H_2_O_2_ to form 2′,7′-dichlorofluorescein (DCF). Cells were treated as described above. 10 μM H_2_DCFDA in PBS was sequentially added and incubated for 30 min in the dark. ROS formation was detected using the microplate reader at excitation 492–495 nm and emission 517–527 nm.

### Western Blot Analysis

After the treatment, cells were collected and lysed by 1X RIPA buffer (Cell Signaling Technology, USA). Total proteins were extracted and quantified the amount of protein by the Bradford assay. Following the electrophoresis, the separated proteins were electrotransferred from the gel to polyvinylidene difluoride (PVDF) membranes. These were blocked with 5% non-fat milk in Tris-buffer containing 0.1% Tween-20 for 2 h, followed by incubation with primary antibodies including BAX, BCL-2, SIRT1, SIRT3, FOXO3a, CAT, SOD2, and actin at 4°C overnight. Subsequently, the blots were incubated with the anti-rabbit or anti-mouse IgG-HRP at room temperature for 1 h. The protein signals were visualized with a ChemiDoc™ MP Imaging System (Bio-Rad, Hercules, CA, USA).

### Immunofluorescence

In order to observe the presence/absence and localization of the selected proteins, fluorophore-tagged antibodies were utilized. After the treatment, cells were fixed in 4% paraformaldehyde and permeabilized with 1% Triton-X 100 in PBS. Non-specific targets were blocked by 1% bovine serum albumin (BSA), followed by incubation with anti-SIRT1 antibody and Alexa Fluor 488-conjugated goat anti-mouse IgG. Following PBS washing, the slides were mounted and examined under an Olympus FluoView FV1000 confocal microscope (Olympus, Tokyo, Japan).

### Computational Modeling

Molecular docking analysis was performed to investigate the molecular interaction of alpha-mangostin and memantine with the activator-binding site of SIRT1 using AutoDock 4.2.6 program (22). Crystallographic structures of SIRT1 complexed with resveratrol were obtained from RCSB Protein Data Bank (PDB code 5BTR). Atomic coordinates of alpha-mangostin and memantine were obtained from PubChem database (http://pubchem.ncbi.nlm.nih.gov). Before doing the docking calculation, polar hydrogen atoms were added into the protein and tested compounds. Rotational bonds of the protein structure were considered as rigid, and those of the compound structures were treated as flexible. Docking parameters of a Lamarckian genetic algorithm were performed for 100 runs (23). A grid box size was set to cover the interface between the catalytic domain (CD) and N-terminal domain (NTD) of SIRT1. The docking results were analyzed and visualized the interactions between each compound and SIRT1 enzyme by Discovery Studio Visualizer 2016 (Accelrys, Inc., San Diego, CA).

### Statistical Analysis

One-way analysis of variance (One-way ANOVA), followed by a Tukey-Kramer test, was used to determine the differences between the untreated and treated groups using GraphPad Prism 6 (GraphPad Software Inc., CA, USA). The data from the three independent biological replicates were used for the statistical testing. A value of *P* < 0.05 was considered statistically significant.

## Results

### Cytotoxic Effect of Alpha-Mangostin Against H_2_O_2_-Induced Neurotoxicity

Exposure of neuronal SH-SY5Y cells to increasing concentrations of alpha-mangostin and memantine was assessed by MTT assay. As shown in Figure 1B, cells treated with 1 and 5 μM alpha-mangostin had no toxic effect on cellular viability in SH-SY5Y cells. The higher concentrations of alpha-mangostin (10 and 20 μM) reduced cell viability compared with the untreated cells by more than 50%, which are consistent with previous reports (24, 25). Moreover, cell viability remained unchanged until 10 μM memantine, but significantly decreased when the cells were treated with 20 μM memantine compared with the control (Figure 1C). Previously, it was determined that H_2_O_2_ resulted in a loss of cell viability in SH-SY5Y cells at concentrations above 400 μM, so in this work, H_2_O_2_, one of the most stable ROS, was used at 400 μM to induce oxidative injury in the cell-based model (26, 27). Surprisingly, alpha-mangostin-enriched neuronal cells exposed to 400 μM H_2_O_2_ significantly retained viability similar to that of the memantine-enriched cells at the concentration of 1 μM (Figure 1D). Therefore, 1 μM alpha-mangostin and memantine were adopted as the optimal concentration for examining H_2_O_2_ effects in the subsequent experiments. As detected under a light microscope, apoptotic features were observed in SH-SY5Y cells after treatment with 400 μM H_2_O_2_ exhibiting cell shrinkage, no longer adherent, and membrane blebbing. However, the number of dead cells was less in alpha-mangostin or memantine pretreatment than the untreated cells, and the occurrence of apoptotic cell death was mitigated in H_2_O_2_-treated cells (Figure 1E).

### Inhibitory Effect of Alpha-Mangostin on H_2_O_2_-Induced Apoptotic Cascades in SH-SY5Y Cells

**Figure 2:**
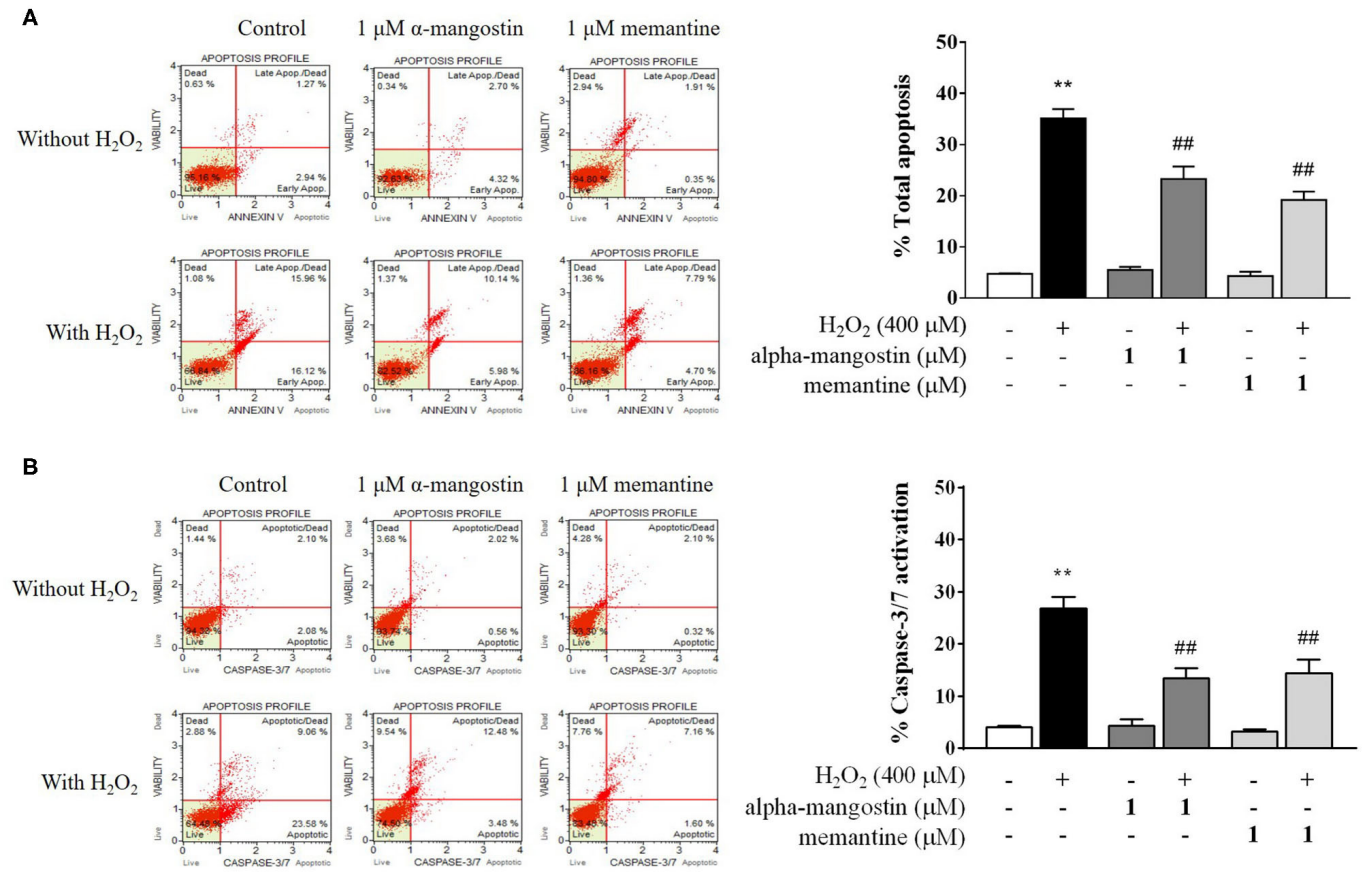
Evaluation of apoptotic profiles of SH-SY5Y cells pretreated with alpha-mangostin and memantine against H_2_O_2_ exposure using the Muse Cell Analyzer. Representative scatter plots of live (lower left), early apoptotic (lower right), late apoptotic (upper right), and dead cell (upper left) were measured by assay of **(A)** Annexin/7-AAD and **(B)** Caspase-3/7 activation. Early and late apoptotic rates were presented as percentages of total apoptosis. Data are presented as mean ± SEM of four independent experiments. **P < 0.01 vs. control and ^*##*^*P* < 0.01 vs. H_2_O_2_.

Apoptotic profiles of alpha-mangostin and memantine against H_2_O_2_ treatment were investigated by annexin V/7-AAD assay (Figure 2A). The percentage of cells with apoptotic cell population (annexin V positive/7-AAD negative and annexin V positive/7-AAD positive) in the H_2_O_2_-induced cell death was significantly higher than that of the untreated group (35.14%). There were no statistically significant differences in the group of cells treated with 1 μM alpha-mangostin or memantine alone compared with the control. The pretreatment of alpha-mangostin or memantine decreased apoptotic-like cells due to H_2_O_2_ treatment; 23.29 % and 19.20 %, respectively.

Caspase-3/7 activation was further confirmed the effect of pretreatment with 1 μM alpha-mangostin and memantine on apoptotic cascades (Figure 2B). Apoptotic cells exhibiting caspase-3/7 activity (caspase-3/7 positive/7-AAD negative) and late apoptotic/dead cells (caspase-3/7 positive/7-AAD positive) were presented as percentages of total caspase-3/7 activation. After being treated with H_2_O_2_ as described, the percentage of caspase-3/7 activation markedly increased to 26.85% compared with the control. The result demonstrated that the concentration of 400 μM H_2_O_2_ damaged cells by triggering the induction of cell death. Compared with the control group, alpha-mangostin or memantine itself had no effect on sequential executioners of apoptosis in SH-SY5Y cells, as assessed by caspase-3/7 activation. Particularly, both alpha-mangostin and memantine significantly decreased the caspase-3/7 activation (13.41% and 14.33%, respectively) compared with the H_2_O_2_-treated group.

**Figure 3:**
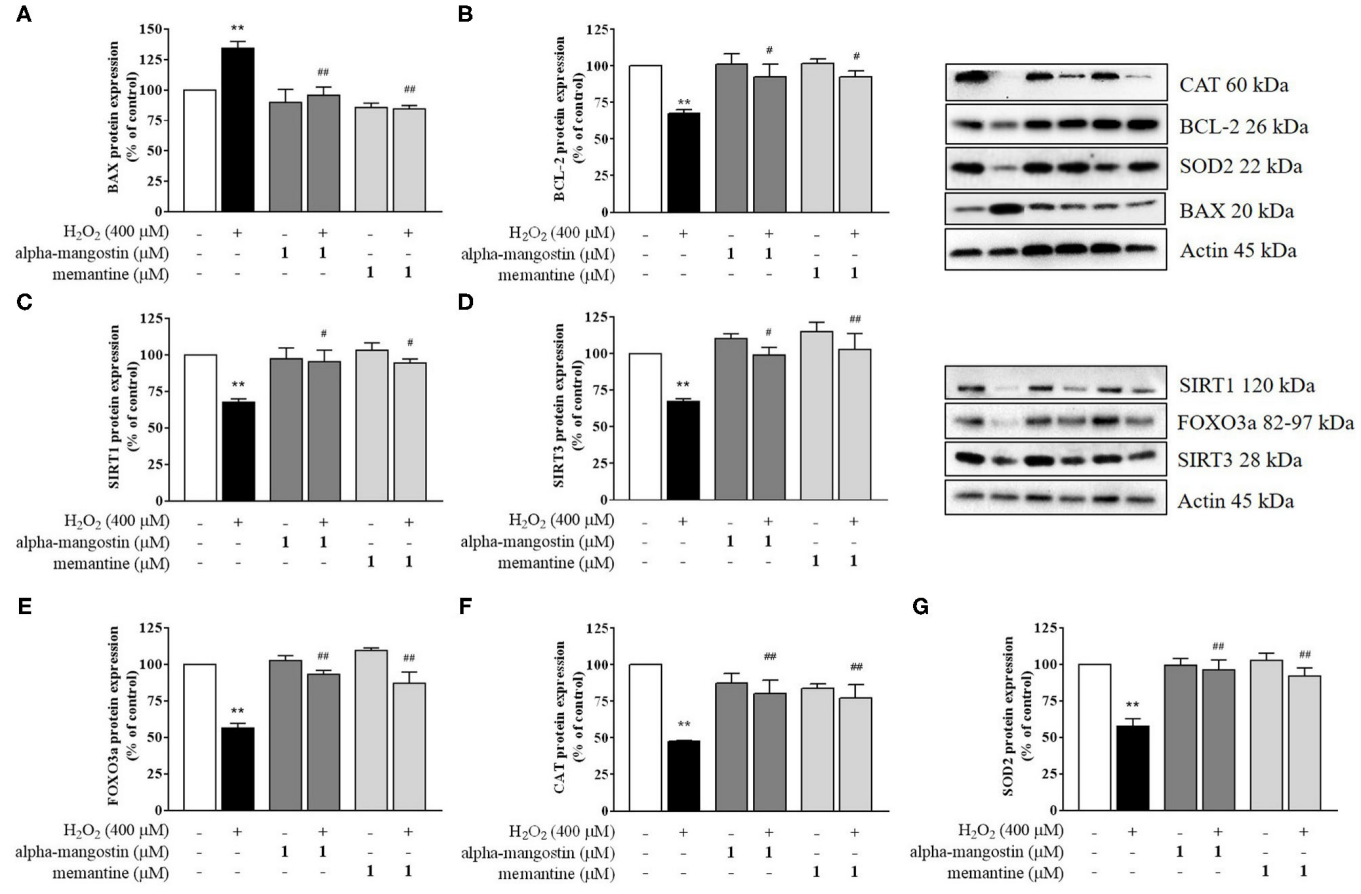
Regulation of sirtuin by alpha-mangostin attenuated H_2_O_2_-induced neurotoxicity in SH-SY5Y cells. The cells were pretreated with/without 1 μM alpha-mangostin and memantine for 3 h prior to H_2_O_2_ exposure. The protein expressions of **(A)** BAX, **(B)** BCL-2, **(C)** SIRT1, **(D)** SIRT3, **(E)** FOXO3a, **(F)** CAT, and **(G)** SOD2 were determined by Western blot. Data are presented as mean ± SEM of three independent experiments. **P < 0.01 vs. control and ^#^*P* < 0.05, ^*##*^*P* < 0.01 vs. H_2_O_2_.

Apoptosis is a terminal cellular event that is promoted by the BCL-2 superfamily of cell death regulators, such as BAX and BCL-2, which initiate the caspase cascade and eventually result in cell death via enzymatic destruction of cytoplasmic proteins and DNA (28, 29). To further investigate the mechanism of alpha-mangostin on oxidative stress, protein expressions of the BCL-2 family were elucidated by Western blot analysis. As shown in Figures 3A,B, H_2_O_2_ significantly increased BAX and decreased BCL-2 protein expressions. Treatment with 1 μM alpha-mangostin and memantine did not significantly alter positive and negative regulators of apoptosis like BCL-2 family. However, alpha-mangostin showed an ability to protect neuronal cells against H_2_O_2_ treatment by reducing BAX and inducing BCL-2, similar to memantine. These results suggested that pretreatment with alpha-mangostin or memantine shifted the balance between pro- and anti-apoptotic factors toward cell survival.

### Alpha-Mangostin Is Involved in Neuroprotection by Activating SIRT1/3 Signaling Pathway

A possible mechanism of alpha-mangostin on H_2_O_2_-induced oxidative damage in SH-SY5Y cells is through the regulation of cell survival. Thus, activation of the sirtuin family in neuronal cells by pretreatment with alpha-mangostin and memantine were investigated in depth. Exposure to H_2_O_2_ caused a reduction of SIRT1 and SIRT3 protein expression compared with the untreated cells (Figures 3C,D). alpha-Mangostin did not cause any significant changes in the levels of both detected proteins similar to memantine. Interestingly, pretreatment with 1 μM alpha-mangostin or memantine rescued the protein expressions of SIRT1 and SIRT3 under oxidative stress.

**Figure 4:**
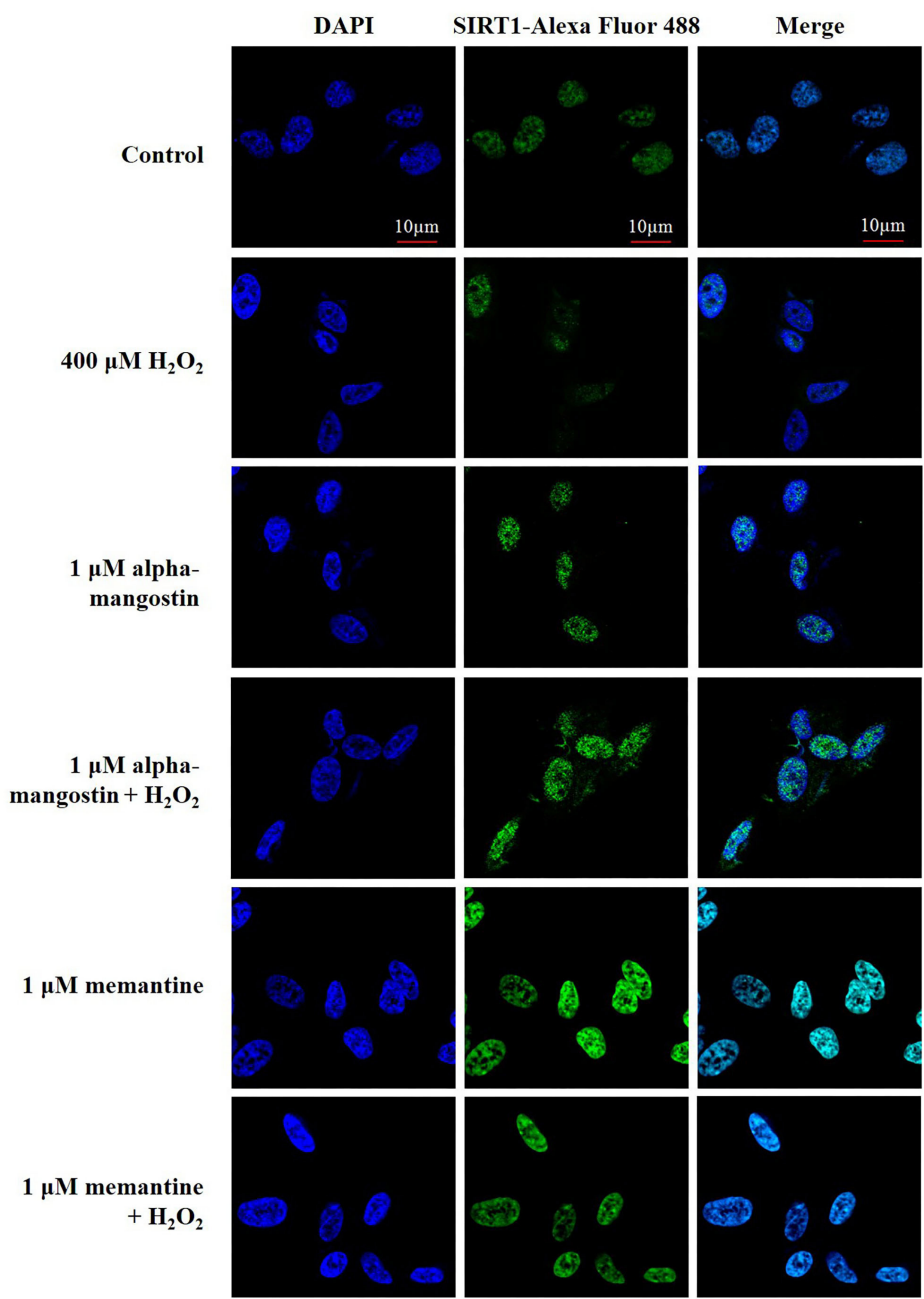
The expression and nuclear translocation of SIRT1 increased along with the pretreatments of 1 μM alpha-mangostin and memantine in SH-SY5Y cells after the oxidative damage. Double immunofluorescence staining with antibody against SIRT1 (green) and DAPI (blue) of the cells was performed. Scale bars = 10 μm.

Additionally, the expression and localization of SIRT1 protein were further confirmed by immunofluorescence staining. Cells were stained with DAPI to visualize nuclei. Imaging stain using SIRT1-Alexa Fluor 488 showed that the green fluorescence intensity induced by H_2_O_2_ was markedly less than that of the cells in the control. Consistent with the above results, fluorescence microscopy data also confirmed that pretreatment with alpha-mangostin or memantine was associated with higher fluorescence intensity in the nucleus compared with that found in the H_2_O_2_-treated group (Figure 4).

Mitochondria play an important role in converting food dietary into cellular energy for biological processes. On the other hand, they enable cells to maintain homeostasis in human body including ATP production, innate immunity regulation, calcium balance, programmed cell death, and stem cell regulation. SIRT1 and SIRT3 influence mitochondrial function by increasing membrane potential, reducing reactive oxygen species (ROS) production, and balancing antioxidant hemostasis (17–19). Of interest, SIRT1 and SIRT3 also regulate the forkhead transcription factor FOXO3a through the control of necessary genes for cell death, and the level of FOXO3a was significantly decreased after H_2_O_2_ treatment. The results showed that alpha-mangostin can activate FOXO3a protein expression comparable to the effect of memantine (Figure 3E). Therefore, mitochondrial deficits associated with neurodegeneration might be delayed or even prevented by alpha-mangostin and memantine through SIRT3-FOXO3a activation.

### Effect of Alpha-Mangostin on H_2_O_2_-Induced Oxidative Stress in SH-SY5Y Cells

**Figure 5:**
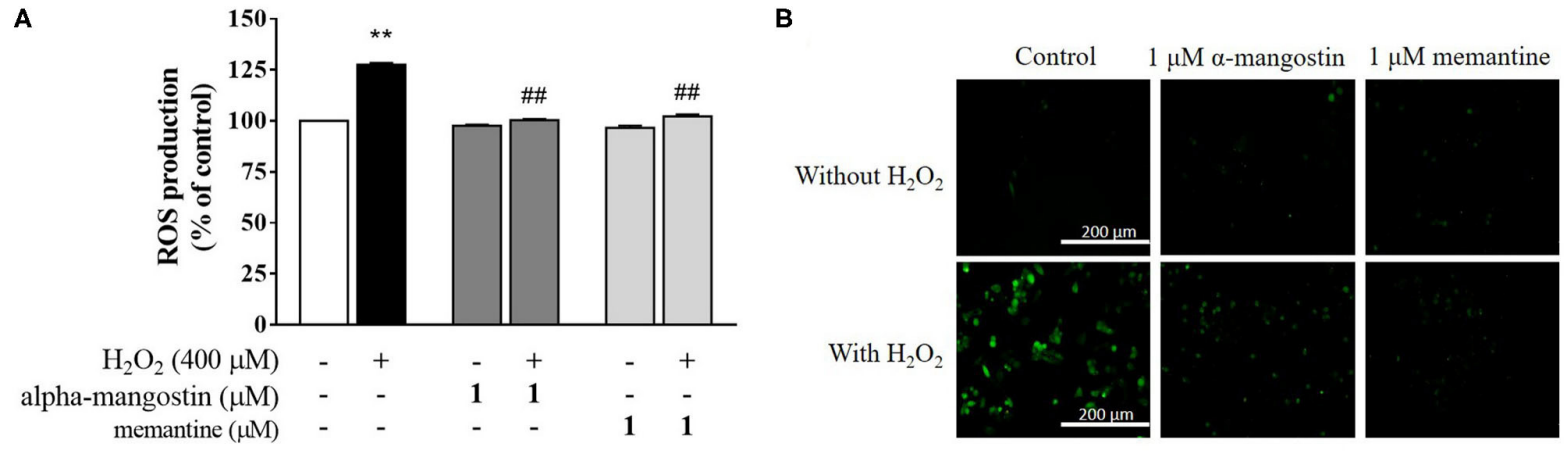
alpha-Mangostin decreased H_2_O_2_-induced ROS generation. **(A)** Cells were pretreated with/without 1 μM alpha-mangostin and memantine for 3 h prior to H_2_O_2_ treatment. The intracellular ROS levels were determined by H_2_DCFDA assay. **(B)** Representative fluorescence microscopy image of the cells stained with H_2_DCFDA was observed by fluorescence microscope. Scale bars = 200 μm. Data are presented as mean ± SEM of four independent experiments. **P < 0.01 vs. control and ^*##*^*P* < 0.01 vs. H_2_O_2_.

In order to determine whether alpha-mangostin or memantine possesses antioxidant property in H_2_O_2_-treated neuronal cells, H_2_DCFDA was applied as an indicator of intracellular ROS generation. As expected, an increase in intracellular ROS with 400 μM H_2_O_2_ was achieved in the cells compared with the untreated cells (Figure 5A). No significant differences in the ROS formation were identified in the cells of control and alpha-mangostin or memantine treatment. Interestingly, pretreatment with 1 μM alpha-mangostin significantly reduced the H_2_O_2_-induced ROS generation from 127.5% to 100.4% comparable to the effect of memantine 102.2%. Consistent with the above results, imaging stain using H_2_DCFDA showed that the green fluorescence intensity induced by H_2_O_2_ was dramatically stronger than the control. However, the intensity was reduced by pretreatment with either alpha-mangostin or memantine, indicating that alpha-mangostin and memantine attenuated the ROS production (Figure 5B).

In addition, SIRT1 and SIRT3 play critical roles in preventing apoptosis by balancing antioxidant defenses and inhibiting the components of MPTP. The changes observed in ROS formation led us to further analyze intracellular antioxidant enzymes, specifically CAT and SOD2 protein expressions. The protein expressions of CAT and SOD2 exposed to 400 μM H_2_O_2_ were significantly lower than those of the control, a finding consistent with the lack of antioxidant responses (Figures 3F,G). alpha-Mangostin at the concentration of 1 μM did not cause any significant changes of CAT and SOD2 in the cells as well as memantine. In contrast, CAT protein expression decreased by H_2_O_2_ treatment was partially restored by pretreatment with alpha-mangostin or memantine (Figure 3F). Similarly, the protein expression of SOD2 significantly enhanced in H_2_O_2_-treated cells which were pretreated with alpha-mangostin or memantine compared with the H_2_O_2_ alone (Figure 3G). Altogether, these results indicated that alpha-mangostin and memantine facilitated the maintenance of CAT and SOD2 enzyme activity in the cells during oxidative stress.

### Prediction of Alpha-Mangostin Binding to the Activator-Binding Sites of SIRT1

**Figure 6:**
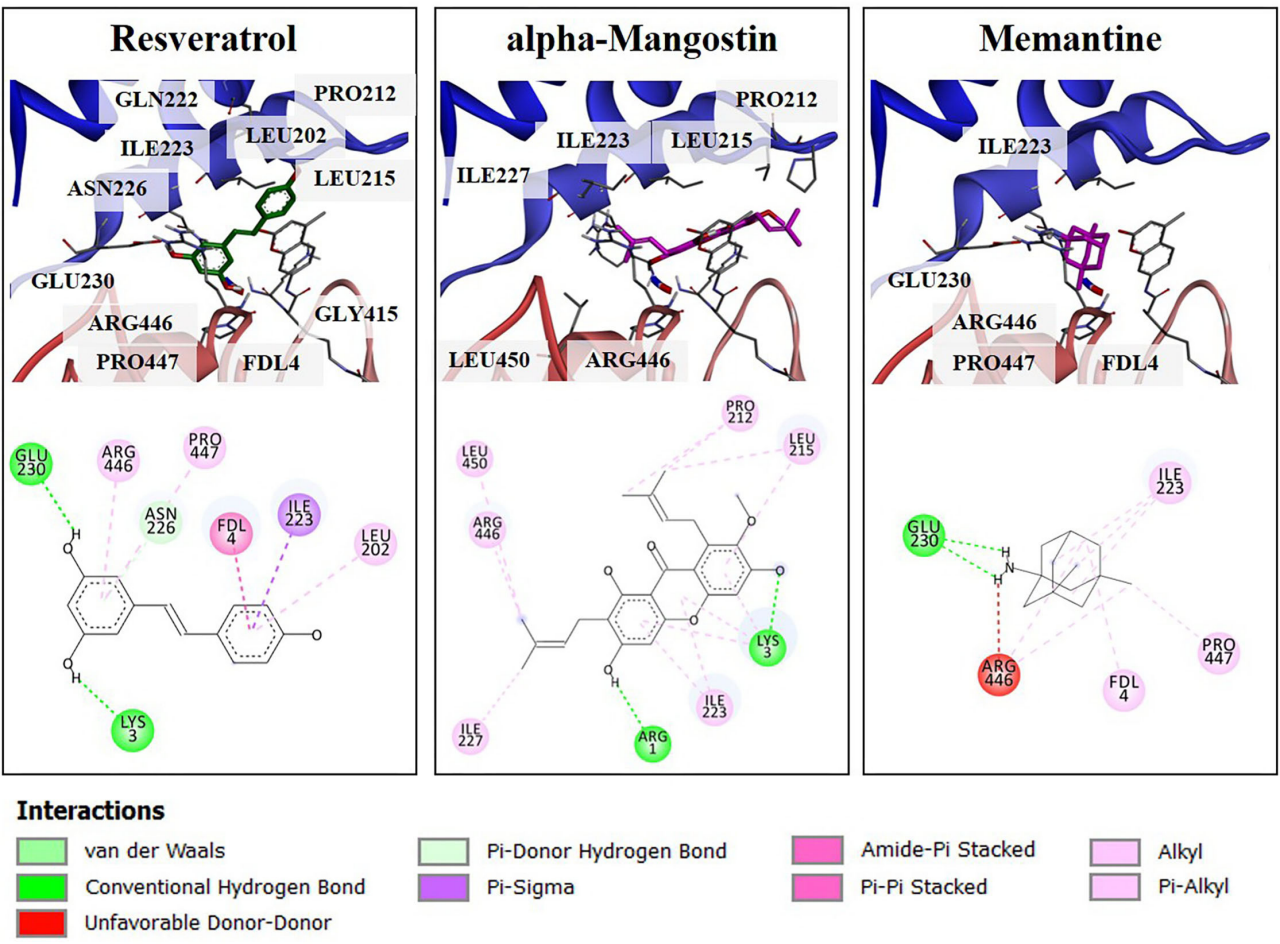
The 3D illustrations of different binding modes of resveratrol, alpha-mangostin, and memantine in SIRT1 activator binding site (PDB code 5BTR). The amino acid residue (carbon colored gray), resveratrol (carbon colored green), and compound interest (carbon colored magenta) are shown in a stick model. The 2D structure of compounds was drawn in a black line. The interaction of residues was represented as a colored ball according to different types of interactions.

To obtain more understanding of the protective effects of alpha-mangostin and memantine to the target SIRT1 [PDB code 5BTR (27, 30)], molecular interaction was further performed by Autodock. Resveratrol is a polyphenol, mainly contained in grape seeds. It was initially identified as an activator of SIRT1 through *in vitro* and *in vivo* screenings. Besides, resveratrol was utilized as a reference small molecule to modulation of SIRT1 activity in *in silico* studies. As shown in Figure 6, several residues surrounding the active site of SIRT1 were shown to form several hydrogen bonds and π-type interactions with the alpha-mangostin. These residues included ILE223, LEU215, and PRO212 located on NTD and ARG446 on CD-binding site of SIRT1, which contributed to SIRT1-resveratrol interactions and made the contacts with alpha-mangostin. Similarly, the catalytic residues (ARG446, GLU230, and PRO447) and the N-terminal residue (ILE223) interacted with SIRT1-memantine by forming both types of bonding. Assessment of molecular docking on the SIRT1 activator binding sites indicated that both alpha-mangostin and memantine could be situated in the same protein binding site of resveratrol. Furthermore, the estimated binding free energies of alpha-mangostin and memantine were −9.31 and −7.49 kcal/mol, respectively, compared with the resveratrol (−7.57 kcal/mol). These results revealed that the complexed compound-SIRT1 for alpha-mangostin was more stable than resveratrol or memantine-SIRT1.

## Discussion

Neuroscience research was mostly conducted in primary neuronal cultures from animal models; however, all of these models show lack of sufficient human identity, short propagation, time and cost-consuming. Currently, drug candidates and toxicity screening processes rely on the *in vitro* cell-based assays, allowing the entire libraries of potential pharmacologically relevant or possible toxin molecules to be screened for cell signaling pathways relevant to therapeutic goals. To counteract this, many neuron-like cells have been performed, resulting in partial expression of neuronal features and human mechanistic information. One of the most popular *in vitro* models is human SH-SY5Y neuroblastoma cell according to its neuroblast-like characteristic, it has been widely used for studying protective effects and underlying mechanisms of bioactive compounds on neuronal cells induced by oxidative damage (31, 32). Oxidative damage plays a crucial role in complications of functional decline in aging individuals. The over-accumulation of ROS generated by both extrinsic and intrinsic sources is initiated within the mitochondria under some stress conditions. Along with the absence of effective antioxidant defenses, this imbalance contributes to the probability of developing neurodegeneration. Exogenous H_2_O_2_ is one of the major causes of oxidative damage. Several lines of research have investigated the efficacy of bioactive compounds under H_2_O_2_-induced stress conditions based on the culture of human neuroblastoma SH-SY5Y cells to assess their antioxidative and antiapoptotic property (33–36). This data is in agreement with the result described for the cytotoxic effect. 400 μM H_2_O_2_ was diffusible through the membrane to significantly stimulate intracellular ROS levels and activate cytotoxicity in SH-SY5Y cells. Present findings are interesting given the potential effect of alpha-mangostin under oxidative stress in human neuronal cells. The neuroprotection conferred by alpha-mangostin could most likely be enhanced via caspase modulation that ends in the executioner caspases-3 and−7. In addition, this could be due to the expression of cell survival signaling like the BCL-2 family. Pretreatment with alpha-mangostin downregulated pro-apoptotic BAX and restored anti-apoptotic BCL-2 which inhibit the formation of MPTP, block the release of cytochrome c, and ultimately suppress the caspase cascade (28–31).

Sirtuin family is a well-known group of cellular survival proteins related to neuroprotection. Changes in the expression and activity of the sirtuin are linked to an age-associated process which regulates cellular metabolic functions impacting on cell survival, oxidative phosphorylation, and maintenance of ATP levels (37). Surprisingly, alpha-mangostin directly upregulated the expression of SIRT1/3 proteins as well as FOXO3a. These findings are consistent with the previous investigation demonstrating interactions between alpha-mangostin derivatives and SIRT1 protein expression under a condition of aging. alpha-Mangostin induces cellular senescence in high-glucose conditions to prevent cardiovascular complications, which is probably due to its antioxidant activity through the SIRT1-AMPK pathway (38). Alpha-mangostin also modulates anti-inflammatory responses as COX-2 and iNOS via the activation of SIRT1-NF-κB in human monocytes (39). Xanthone derivative, gamma-mangostin, protects hepatocytes from acute liver injury by deacetylated SIRT1, enhanced NRF2, and upregulated PGC-1α to promote mitochondrial function in carbon tetrachloride-induced mice (40). In addition to our *in vitro* experiments, we further investigated the natural bioactive compound (alpha-mangostin) and the SIRT1 activator-binding site using the molecular docking approach. Crystallographic and biochemical data have documented that NTD-bound resveratrol promotes tight binding between SIRT1 and the fluorophore-attached peptide, as well as the stimulation of SIRT1 activity (30). With the reference compound resveratrol, it was found that, regardless of the nature of the substrates, the stimulatory effect requires the presence of NTD in addition to the CD of SIRT1. As expected, alpha-mangostin could be docked well into the CD and NTD of SIRT1. Therefore, The SIRT1/3-FOXO3a axis is a conserved cell survival pathway that regulates oxidative damage.

Under the stimulus of antioxidant and oxidative stress, the activated SIRT1 translocated into the nucleus and SIRT3 localized to the mitochondria interact with FOXO3a transcription factor to detoxify ROS levels through upregulation of endogenous antioxidant enzymes as well as the protection of DNA and modulation of cell apoptosis (41, 42). Phenolic compounds can upregulate SIRT1 and FOXO transcription factors as well as induce the antioxidant enzymes and BCL-2 anti-apoptotic protein against H_2_O_2_-induced toxicity in SH-SY5Y cells (27). alpha-Mangostin has been reported to scavenge ROS against mitochondrial toxin 3-nitropropionic acid in primary cerebellar neurons by increasing SOD2 and CAT protein expression (24). In agreement with previous studies, pretreatment with alpha-mangostin recovered the downstream target of the SIRT1/3-FOXO3a pathway, endogenous antioxidant enzymes. Both CAT and SOD were upregulated by alpha-mangostin against H_2_O_2_-induced oxidative stress. This may be explained by the antioxidant capacity of alpha-mangostin related xanthone derivatives. The position and the number of hydroxyl groups in the scaffold influence their inhibition of lipid peroxidation, scavenging activity, and metal-chelating capacity (43). It is capable of protecting cell death by forming directly bonds with free radicals, neutralizing, or converting them into more stable and less reactive molecules (44).

Our oxidative stress model provides an important view-point based on the NMDA receptor antagonist (memantine) which is the fourth FDA-approved treatment of AD. Specifically, memantine reduces the release of LDH in neuronal cells in the absence of Aβ_1−42_, suggesting that overproduction of ROS may exert toxicity via the NMDA receptor leading to age-induced cognitive deficits (45, 46). However, the impact of NMDA receptor antagonism on sirtuin is limited. When H_2_O_2_-induced oxidative condition, memantine was determined the biological properties through activation of SIRT1/3-FOXO3a signaling pathway compared with alpha-mangostin. In the MTT assay, through annexin V/7-AAD staining and caspase-3/7 activation, memantine was confirmed for its anti-apoptotic activity. Through the western blot on equal terms, the activation of SIRT1/3 related downstream targets including FOXO3a, CAT, and SOD was improved by regulation of memantine. In support of the antioxidant and protective actions, the molecular interaction of SIRT1-memantine was further investigated compared with the resveratrol. The SIRT1 activator binding sites indicated that memantine could be docked well into the same binding site of resveratrol, but the stability was less than alpha-mangostin. Furthermore, conjugation of memantine and ferulic acid modulates NMDAR-mediated excitotoxicity, responsible for Aβ neurotoxicity and neuronal cell death caused by accumulation of ROS (47). Based on this evidence, an alpha-mangostin-memantine complex might trigger NMDAR-mediated neurotoxic events, not only a symptomatic medication but also suppress the progression of AD. Due to the limitation of *in vitro* models, the neuronal behaviors are limited, including the cell-cell interaction, tissue architecture, and intracellular microenvironments. To explore the realistic functions of alpha-mangostin, *in vivo* studies are still needed to be experimentally validated the antioxidant is with respect to pharmacokinetics in the body before clinical trials.

## Conclusion

**Figure 7:**
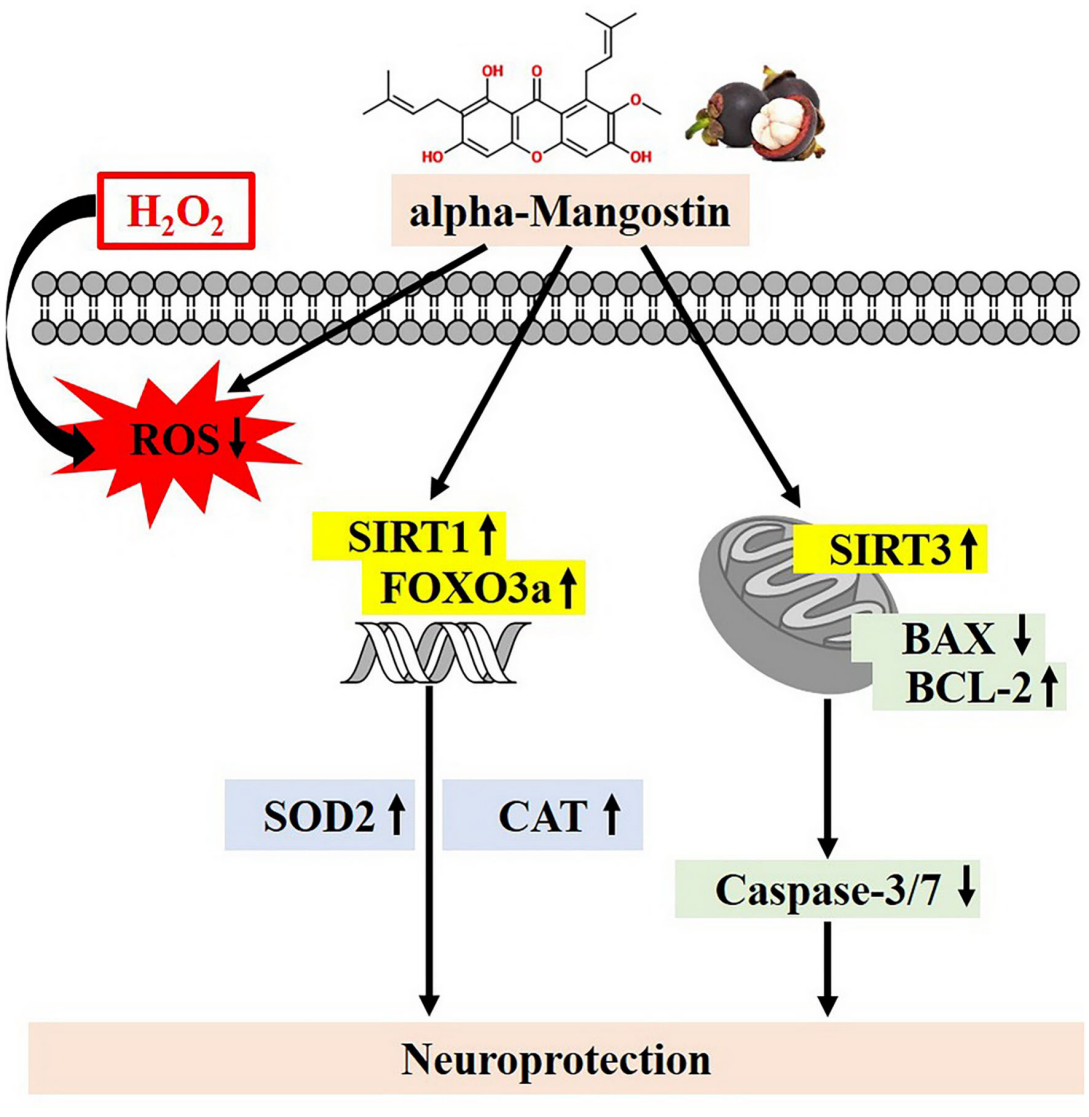
Proposed schematic neuroprotective mechanisms of alpha-mangostin under oxidative damage. alpha-Mangostin attenuates H_2_O_2_-induced apoptosis and oxidative stress in SH-SY5Y neuroblastoma cells via the activation of SIRT1/3-FOXO3a signaling pathway.

To our knowledge, this is the first report describing the protective effects of alpha-mangostin through SIRT1/3-FOXO3a pathway in neuronal cells. alpha-Mangostin significantly attenuated H_2_O_2_-induced neurotoxicity by balancing of antioxidant defenses, activation of the SIRT1/3-FOXO3a, and modulation of mitochondrial functions leading to anti-apoptosis (Figure 7). Taken together, alpha-mangostin might be an adjunctive to conventional treatment or prevention of AD in the future.

## Data Availability Statement

The original contributions presented in the study are included in the article/Supplementary Material, further inquiries can be directed to the corresponding author.

## Author Contributions

WS, KP, and NS conducted the investigation and the methodology. WR and WS undertook formal analysis. WR, WS, KP, and NS undertook visualization and wrote the original draft. WS, KP, SP, and VP undertook writing, review, editing, and contributed to funding acquisition. All authors contributed to the article and approved the submitted version.

## Funding

This study was supported by the Research and Researchers for Industries (RRI) funded by the Thailand Research Fund (TRF) (MSD58I0147) for WR.

## Conflict of Interest

The authors declare that the research was conducted in the absence of any commercial or financial relationships that could be construed as a potential conflict of interest.

## Publisher's Note

All claims expressed in this article are solely those of the authors and do not necessarily represent those of their affiliated organizations, or those of the publisher, the editors and the reviewers. Any product that may be evaluated in this article, or claim that may be made by its manufacturer, is not guaranteed or endorsed by the publisher.
