# Supplementary material for: Modulatory Effects of Alpha-Mangostin Mediated by SIRT1/3-FOXO3a Pathway in Oxidative Stress-Induced Neuronal Cells
Source: Front Nutr. 2022 Jan 28;8:714463. doi: 10.3389/fnut.2021.714463 (PMC8835347; doi:10.3389/fnut.2021.714463)
Supplement: Supplementary file 1 [file Data_Sheet_1.PDF]

Supplementary Material

1. Supplementary Figures 3 Once the gel has been transferred to a blot, a razor blade and ruler were used to cut the blot into strips. The strips can then be incubated with multiple antibodies. The intensity of protein band was visualized and analyzed using ChemiDoc™ MP Imaging System with Image Lab program. Image processing methods (brightness, contrast, cropping, and labeling) were applied by Microsoft PowerPoint 2016 64-Bit Edition.

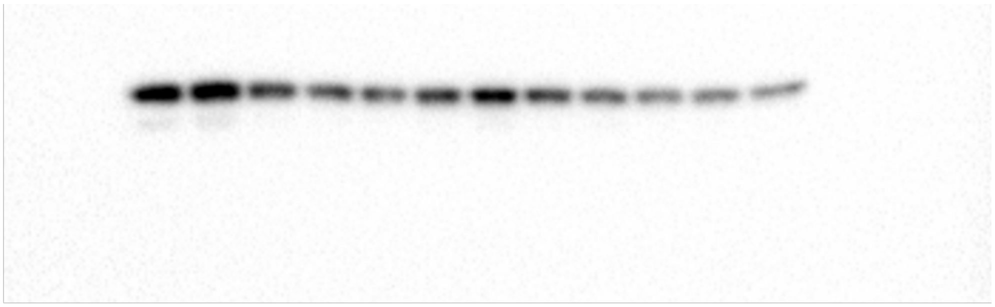

H_2O_2	-	+	-	+	-	+	-	+	-	+	-	+	Blank	Marker
Compound A	-	-	+	+	-	-	-	-	-	-	-	-		
Compound B	-	-	-	-	+	+	-	-	-	-	-	-		
Compound C	-	-	-	-	-	-	+	+	-	-	-	-		
Alpha-mangostin	-	-	-	-	-	-	-	-	+	+	-	-		
Memantine	-	-	-	-	-	-	-	-	-	-	+	+		

Supplementary Figure 3A. The protein expressions of BAX were determined by Western blot.

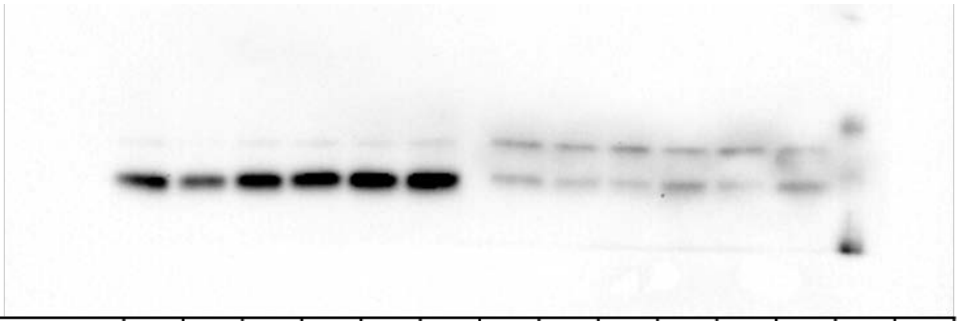

H_2O_2	-	+	-	+	-	+	-	+	-	+	-	+	Marker	Blank
Alpha-mangostin	-	-	+	+	-	-	-	-	-	-	-	-		
Memantine	-	-	-	-	+	+	-	-	-	-	-	-		
Compound A	-	-	-	-	-	-	+	+	-	-	-	-		
Compound B	-	-	-	-	-	-	-	-	+	+	-	-		
Compound C	-	-	-	-	-	-	-	-	-	-	+	+		

Supplementary Figure 3B. The protein expressions of BCL-2 were determined by Western blot.

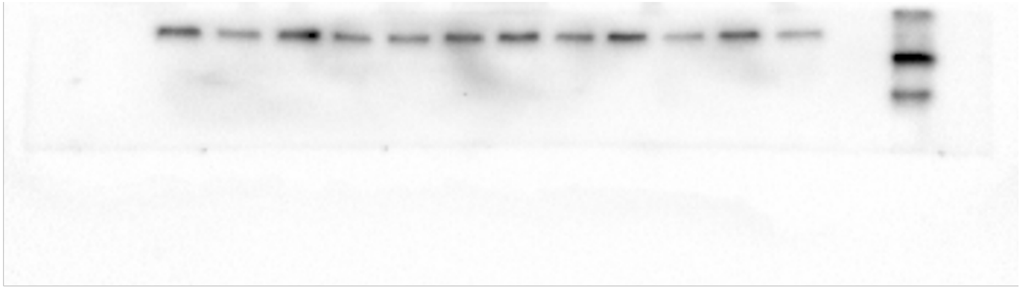

H_2O_2	-	+	-	+	-	+	-	+	-	+	-	+	Blank	Marker
Compound A	-	-	+	+	-	-	-	-	-	-	-	-		
Compound B	-	-	-	-	+	+	-	-	-	-	-	-		
Compound C	-	-	-	-	-	-	+	+	-	-	-	-		
Alpha-mangostin	-	-	-	-	-	-	-	-	+	+	-	-		
Memantine	-	-	-	-	-	-	-	-	-	-	+	+		

Supplementary Figure 3C. The protein expressions of SIRT1 were determined by Western blot.

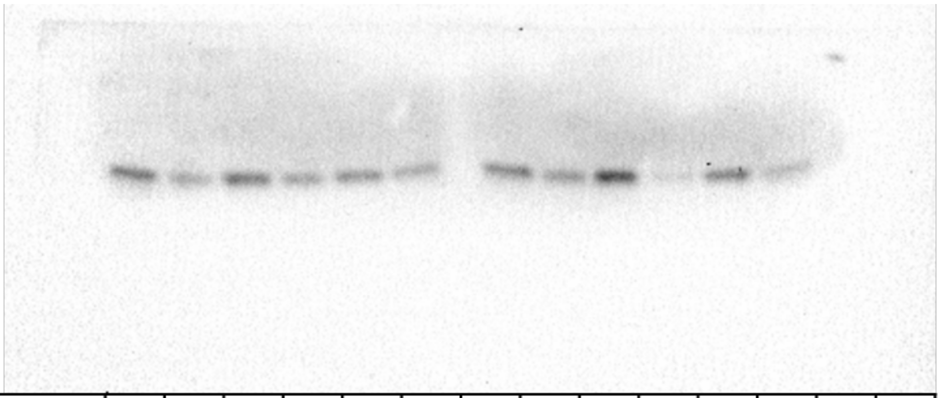

H_2O_2	-	+	-	+	-	+	-	+	-	+	-	+	Marker	Blank
Alpha-mangostin	-	-	+	+	-	-	-	-	-	-	-	-		
Memantine	-	-	-	-	+	+	-	-	-	-	-	-		
Compound A	-	-	-	-	-	-	+	+	-	-	-	-		
Compound B	-	-	-	-	-	-	-	-	+	+	-	-		
Compound C	-	-	-	-	-	-	-	-	-	-	+	+		

Supplementary Figure 3D. The protein expressions of SIRT3 were determined by Western blot.

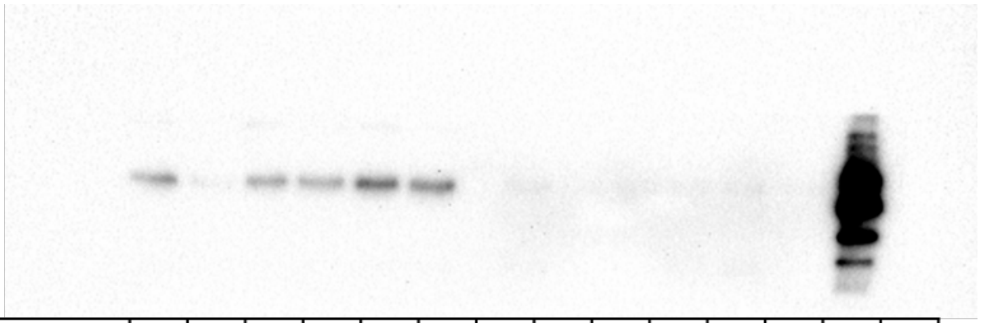

H_2O_2	-	+	-	+	-	+	-	+	-	+	-	+	Marker	Blank
Alpha-mangostin	-	-	+	+	-	-	-	-	-	-	-	-		
Memantine	-	-	-	-	+	+	-	-	-	-	-	-		
Compound A	-	-	-	-	-	-	+	+	-	-	-	-		
Compound B	-	-	-	-	-	-	-	-	+	+	-	-		
Compound C	-	-	-	-	-	-	-	-	-	-	+	+		

Supplementary Figure 3E. The protein expressions of FOXO3a were determined by Western blot.

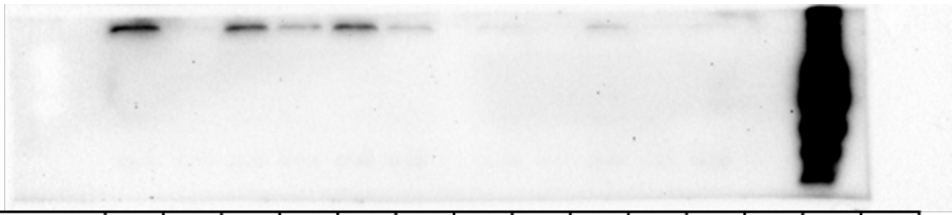

H_2O_2	-	+	-	+	-	+	-	+	-	+	-	+	Marker	Blank
Alpha-mangostin	-	-	+	+	-	-	-	-	-	-	-	-		
Memantine	-	-	-	-	+	+	-	-	-	-	-	-		
Compound A	-	-	-	-	-	-	+	+	-	-	-	-		
Compound B	-	-	-	-	-	-	-	-	+	+	-	-		
Compound C	-	-	-	-	-	-	-	-	-	-	+	+		

Supplementary Figure 3F. The protein expressions of CAT were determined by Western blot.

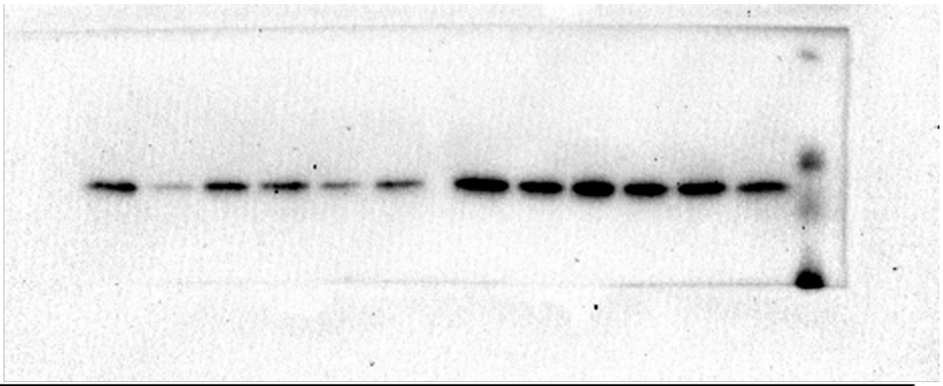

H_2O_2	-	+	-	+	-	+	-	+	-	+	-	+	Marker	Blank
Alpha-mangostin	-	-	+	+	-	-	-	-	-	-	-	-		
Memantine	-	-	-	-	+	+	-	-	-	-	-	-		
Compound A	-	-	-	-	-	-	+	+	-	-	-	-		
Compound B	-	-	-	-	-	-	-	-	+	+	-	-		
Compound C	-	-	-	-	-	-	-	-	-	-	+	+		

Supplementary Figure 3G. The protein expressions of SOD2 were determined by Western blot.

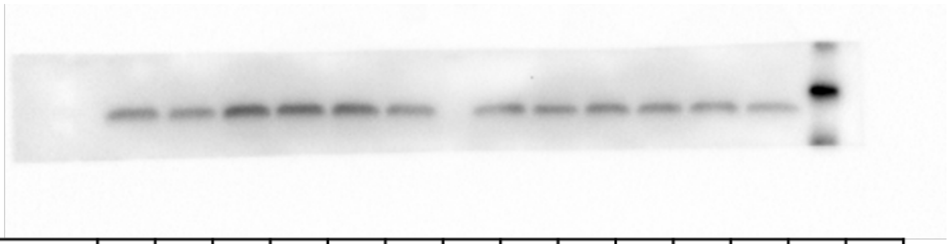

H_2O_2	-	+	-	+	-	+	-	+	-	+	-	+	Marker	Blank
Alpha-mangostin	-	-	+	+	-	-	-	-	-	-	-	-		
Memantine	-	-	-	-	+	+	-	-	-	-	-	-		
Compound A	-	-	-	-	-	-	+	+	-	-	-	-		
Compound B	-	-	-	-	-	-	-	-	+	+	-	-		
Compound C	-	-	-	-	-	-	-	-	-	-	+	+		

Supplementary Figure 3H-1. The protein expressions of actin were determined by Western blot which was the same membrane as CAT, BCL-2, SOD2, and BAX.

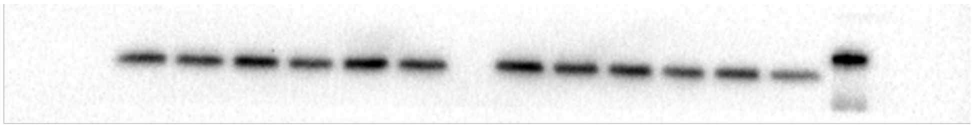

H_2O_2	-	+	-	+	-	+	-	+	-	+	-	+	Marker	Blank
Alpha-mangostin	-	-	+	+	-	-	-	-	-	-	-	-		
Memantine	-	-	-	-	+	+	-	-	-	-	-	-		
Compound A	-	-	-	-	-	-	+	+	-	-	-	-		
Compound B	-	-	-	-	-	-	-	-	+	+	-	-		
Compound C	-	-	-	-	-	-	-	-	-	-	+	+		

Supplementary Figure 3H-2. The protein expressions of actin were determined by Western blot. which was the same membrane as SIRT1, SIRT3, and FOXO3a.
